# New contrast agents for photoacoustic imaging and theranostics: Recent 5-year overview on phthalocyanine/naphthalocyanine-based nanoparticles

**DOI:** 10.1063/5.0047660

**Published:** 2021-07-30

**Authors:** Eun-Yeong Park, Donghyeon Oh, Sinyoung Park, Wangyu Kim, Chulhong Kim

**Affiliations:** 1 Departments of Electrical Engineering, Convergence IT Engineering, Mechanical Engineering, and Medical Device Innovation Center, Pohang University of Science and Technology (POSTECH), Pohang, Gyeongbuk 37673, South Korea; 2 Department of Radiology, School of Medicine, Stanford University, Stanford, California 94305, USA

## Abstract

The phthalocyanine (Pc) and naphthalocyanine (Nc) nanoagents have drawn much attention as contrast agents for photoacoustic (PA) imaging due to their large extinction coefficients and long absorption wavelengths in the near-infrared region. Many investigations have been conducted to enhance Pc/Ncs' photophysical properties and address their poor solubility in an aqueous solution. Many diverse strategies have been adopted, including centric metal chelation, structure modification, and peripheral substitution. This review highlights recent advances on Pc/Nc-based PA agents and their extended use for multiplexed biomedical imaging, multimodal diagnostic imaging, and image-guided phototherapy.

## I. INTRODUCTION

Photoacoustic (PA) imaging is a hybrid imaging modality based on the PA effect that exploits both rich optical contrast and high ultrasonic spatial resolution in deep tissues.^1–4^ It visualizes optical chromophores in biological tissues where ultrasound (US) signals are generated from light absorption and subsequent thermoelastic expansions. In the last decades, PA imaging has been studied extensively, with many advantages for biomedical imaging. First, unlike traditional optical imaging techniques where imaging depth is limited to few millimeters due to round trip optical attenuation in biological tissues, PA imaging can reach up to a few centimeters of depth in biological tissues, thanks to one-way photon propagation and acoustic detection which suffers far much less attenuation and scattering than optical detection.^5–9^ Second, the spatial resolution and achievable imaging depth are scalable based on the US detector frequency, thus covering many applications ranging from organelles and cells to small animals and humans.^10–16^ Third, PA imaging is inherently compatible with many complementary imaging modalities, e.g., conventional US imaging and optical imaging modalities, and thus are highly suitable for multiplexed and multimodal imaging.^17–22^

The PA imaging exploits both endogenous and exogenous contrast agents to image biological tissues. The endogenous agents include DNA/RNA,^23,24^ melanin,^25–28^ oxygenated-/deoxygenated-hemoglobin,^29–31^ lipid,^32–34^ and water.^35^ These agents provide functional, metabolic, and histopathologic information such as blood oxygenation, oxygen metabolism, and vascularity.^36,37^ A large variety of exogenous contrast agents have been investigated, including inorganic nanomaterials (gold nanostructures, carbon nanotubes, etc.), organic dyes (porphyrine-/cyanine-based dyes), and fluorescent proteins [green/red fluorescent protein (GFP/RFP)].^38–42^ Gold nanomaterials are one of the most widely investigated PA agents thanks to their surface plasmon resonance properties and biocompatibilities. The plasmonic peaks can be tuned from the visible to the near-infrared (NIR) range depending on their shapes and provide optical contrast,^43^ but still has limitations for clinical translation due to photoinstability.^44^ Fluorescent proteins have drawn much attention in live-cell optical imaging.^45^ GFP, however, has a lower absorption peak wavelength, high fluorescent quantum yield (i.e., low heat conversion efficiency), and shallow imaging depth for *in vivo* applications.^46^

The phthalocyanine (Pc) and naphthalocyanine (Nc) are organic dyes that have been explored widely for optical imaging and phototherapy due to their strong extinction coefficients and long absorption wavelengths in the NIR region.^47–49^ This region is also known as an optical/biological/therapeutic window where light can penetrate deeply in tissues with low phototoxicity. The Pc/Ncs also have attractive traits of good photostability and tunable optical properties through facile chemical modifications.^50–53^ Low aqueous solubility is the main challenge for Pc/Ncs as a contrast agent, and many attempts have been successfully made to solubilize Pc/Ncs using biocompatible and biodegradable coating (e.g., surfactant modification, micelle/liposome carrier).^54–58^ Several Pc/Nc derivatives, including aluminum Pc, silicon Pc, and zinc Pc, have either already been through the FDA approval process or are under clinical trials.^59,60^

In this review, we summarize the recent advances in Pc/Nc-based nanomaterials as PA imaging agents and synergistic applications for using multimodal imaging and theranostics. First, we present design strategies and processes to enhance the functionality of Pc/Ncs, including photophysical properties and targeting ability. We then focus on their biomedical applications, including contrast-enhanced PA imaging, biosensing of tumor and other biomolecules, and cancer theranostics. Finally, we will discuss future directions for Pc/Nc-based PA agents.

## II. FUNCTIONALIZING P_CS_ AND N_CS_ AS a PHOTOACOUSTIC CONTRAST AGENT

### A. Photophysical property

The two important photophysical properties of nanoparticle for considering them as potential PA contrast agent are: (1) does the peak absorption wavelength ensures sufficient penetrating depth and (2) how the absorbed energy efficiently transfers to thermal expansion, which is directly proportional to photoacoustic signal intensity. These aspects connote two strategies for engineering the nanoparticles; how to tune the wavelength and how to increase the given particle's absorption yield.

#### 1. Absorption spectrum shift

For most biomedical imaging techniques, the allowed penetration depth is under the boundary of the optical ballistic region (< 1 mm). However, the PA imaging crosses over the boundary because the PA technique involves a one-way optical propagation and, independent of scattering, it responds with an acoustic signal. For reaching the maximum penetration depth in tissue, the wavelength of the optical source is generally selected in the specific range of NIR zone (650–1350 nm), named as “NIR window.”^61–63^ In such wavelength range, the photon gets absorbed less by blood than shorter wavelengths and bypasses the absorption by water, compared to longer wavelengths. In addition, determining the absorption wavelength carefully can lead to accurate multiplex imaging or combinatorial therapy such as photodynamic therapy (PDT) or photothermal therapy (PTT).

**FIG. 1.**
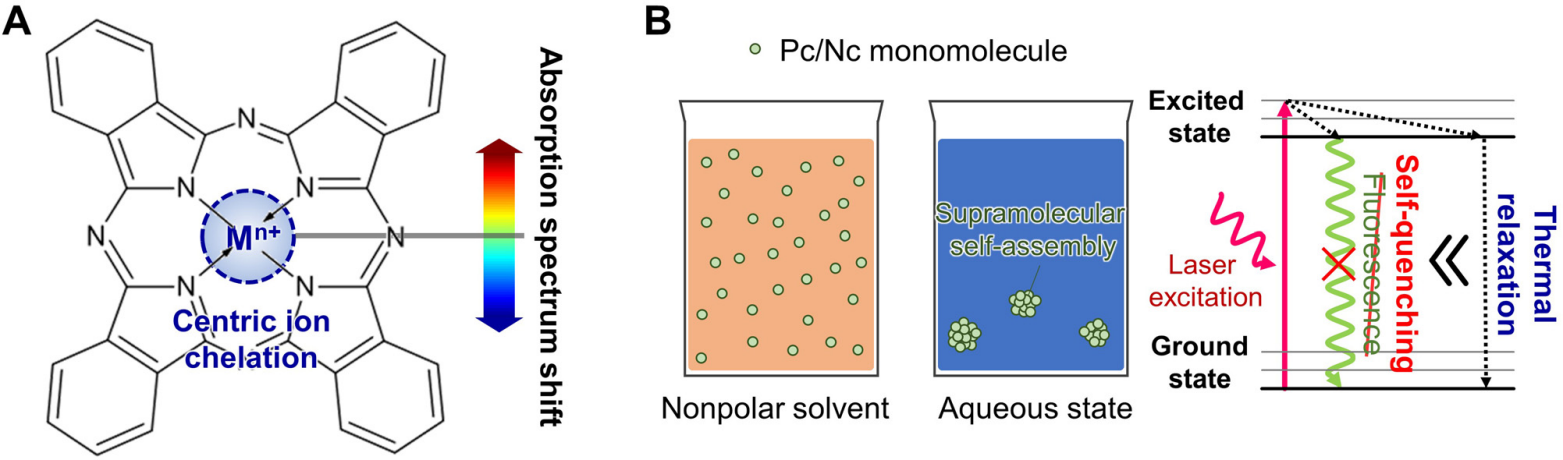
Strategies to functionalize photophysical properties of phthalocyanine/naphthalocyanine. (a) Absorption spectrum shift with centric ion chelation. (b) Increased absorption yield by supramolecular self-assembly in aqueous condition.

Even though the free-base Pc and Nc have their absorption peak around 670 nm and 750 nm in DMF solution, respectively,^64,65^ centric ion chelation, at the center of the Pc/Ncs molecule, enables the tuning of the optical absorption band of the agent [Fig. 1(a)].^66^ Depending on the type of metal, Pc/Nc's absorption peak can be varied through chelation in the range of 650–950 nm. In addition to wavelength tuning, chelation with appropriate diamagnetic metallic ions increases the absorption yield of Pc/Ncs and stabilizes the charge balance, and, consequently, lengthens the molecules' lifetime.^67,68^

Currently, metallic ions such as zinc (Zn),^69^ copper (Cu),^70^ iron (Fe),^71^ and indium (In)^72^ are widely favored in research studies with a corresponding peak absorption of 700, 850, 680, and 840 nm, respectively.

Duffy *et al.* showed through a comprehensive study that Nc particle's central ion change results in maximum PA intensity wavelength shift at 850, 848, 774, and 808 nm, for copper, nickel, silicon, and vanadium ion, respectively.^73^ The study further identified that central ion change also affected singlet oxygen photoproduction and fluorescence pattern.

In addition to metallic ions, variation with nonmetallic ion chelation can also be used for engineering the nanoparticle's optical response. Chelation with semiconducting elements, such as silicon (Si), exhibits good photothermal conversion efficiency and is capable of both PDT and PTT effect,^74^ which enables the use of such material as single-agent theranostic nanoprobes.^58^ Choice of peak absorption to NIR-II window (1000–1700 nm) instead of NIR-I window is advantageous from both aspects: penetration depth and maximum permissible exposure (MPE). Hence, contrast agents with its peak absorption at NIR-II window enlarges the scope of imaging target to deeper organs, which is an important factor to translate from preclinical studies to future clinical application toward human. A number of studies already present the superiority of NIR-II PAI with multi-centimeter penetration depth compared to NIR-I PAI. Together with chicken breast phantom, rodent mammary tumor models, lymphatic mapping, and ultimately with 2.6–5.1-cm-thick compressed human breast, transcendent depth penetrating ability of NIR-II PAI was well validated as well as giving proof to its applicability.^75^ Having deep penetration depth as its major strength, a number of precedential studies eagerly expect its future potential to extend barrier of PAI application toward gastrointestinal tract, lymphatic system, and excretory system.^76^ The electron-deficient group 15 elements (P, As, and Sb) and the electron-rich group 16 elements (S, Se, and Te) can also be used as central substituents, which expands the applicable optical band beyond 1000 nm.^77^ For example, Zhou *et al.* observed intense absorption at 1000 nm with phosphorus Pc to target deeper imaging depth with PACT.^78^ Without using a central inorganic semiconducting element or polymer, Pan *et al.* synthesized NIR-II (1064 nm) agent by arranging four ZnPc molecules into cruciform pentad.^56^

The representative publications for absorption spectrum shift are summarized in Table I.

**TABLE I.** Summary of the representative publications for absorption spectrum shift of phthalocyanine/naphthalocyanine nanaoparticles. $\lambda_{\text{max}}$, maximum absorption wavelength; $\Phi_{F}$, fluorescence quantum yield; FL, fluorescence; $\lambda_{\mathrm{ex}}$, excitation wavelength; $\lambda_{\mathrm{em}}$, emission wavelength; ϵ, molar extinction coefficient; η, photothermal conversion coefficient; $\Phi_{\Delta }$, singlet oxygen generation quantum yield.

Group	Central chelation	$\lambda_{\text{max}}$ (nm)	$\Phi_{F}$	FL $\lambda_{ex}$/ $\lambda_{em}$ (nm)	ϵ (M^−1^ cm^−1^)	Major features
Wang *et al.*^69^	ZnPc	∼700	⋯	650/715	⋯	• η = 31.3 %
						• $\Phi_{\Delta }$ = 0.62
Duffy *et al.*^73^	CuNc	850	0.0003	⋯	2.10 × 10^5^	⋯
	NiNc	848	< 0.02	⋯	1.80 × 10^5^	⋯
	SiNc	774	0.07	⋯	5.70 × 10^5^	⋯
	VNc	808	< 0.02	⋯	2.40 × 10^5^	⋯
Choi *et al.*^70^	CuNc	850	⋯	⋯	⋯	⋯
He *et al.*^71^	Fe(II)Pc	680	⋯	⋯	⋯	⋯
Lobo *et al.*^72^	InPc	840	< 0.001 (at 850 nm)	⋯	9.00 × 10^4^ (at 850 nm)	⋯
Wei *et al.*^58^	SiNc	730	⋯	808/⋯	⋯	• η = ∼59.8 %
Zhou *et al.*^78^	PPc	997	⋯	⋯	6.40 × 10^4^ (at 998 nm)	⋯
Pan *et al.*^56^	Cruciform ZnPc pentad	1040	0.002	880/1108	3.70 × 10^5^	• η = 58.3 %

#### 2. Increased absorption yield

Due to the hydrophobic nature, arising from their π-conjugated planar arrangement, Pc and Nc exhibit high self-aggregation in aqueous solution. During the supramolecular self-assembly phase, collective aggregation induces complete FL self-quenching. It blocks radical oxygen species (ROS) generation pathways, leading to a favorable increase in nonradiative thermal relaxation in its excited phase [Fig. 1(b)].^79,80^ Thus, supramolecular self-assembly can effectively increase PA signal yield compared to its monomolecular application. Liu *et al.* introduced a polymeric nanosystem for near-infrared multispectral PA imaging, which augmented the self-assembly by using hydrophobized ZnPc and oxazoline block copolymer complex (H-PcZn), to efficiently encapsulate the micelles by self-assembly.^81^ Li *et al.* reported a molecular recognition-based supramolecular approach between PcS_4_ and PcN_4_, the two water-soluble Pc derivatives, and observed completely quenched fluorescence (FL) and reduced singlet oxygen generation.^54^

The representative publications for increasing absorption yield are summarized in Table II.

**TABLE II.** Summary of the representative publications for increasing absorption yield of phthalocyanine/naphthalocyanine nanoparticles. $\lambda_{\text{max}}$, maximum absorption wavelength; ϵ, molar extinction coefficient; O.D., optical density; ζ, zeta potential; FL, fluorescence.

Group	Nanomaterial	$\lambda_{\text{max}}$ (nm)	Monomer/assembly size (nm)	ϵ [O.D.]	Major features
Liu *et al.*^81^	Hydrophobized zinc Pc complex	680	−/20–100	8.9 (P-NP);	• Hydrophobizing characterization with oxazoline block copolymer,
				7.4 ( N-NP)	• ζ = P-Pc: +29 mV; N-Pc: −43.8 mV
Li *et al.*^54^	PcS4-PcN4 complex	694	0.5–3/30–100	⋯	• Total FL self-quenching identified at 610 nm

### B. Passive delivery

#### 1. Enhanced solubility

**FIG. 2.**
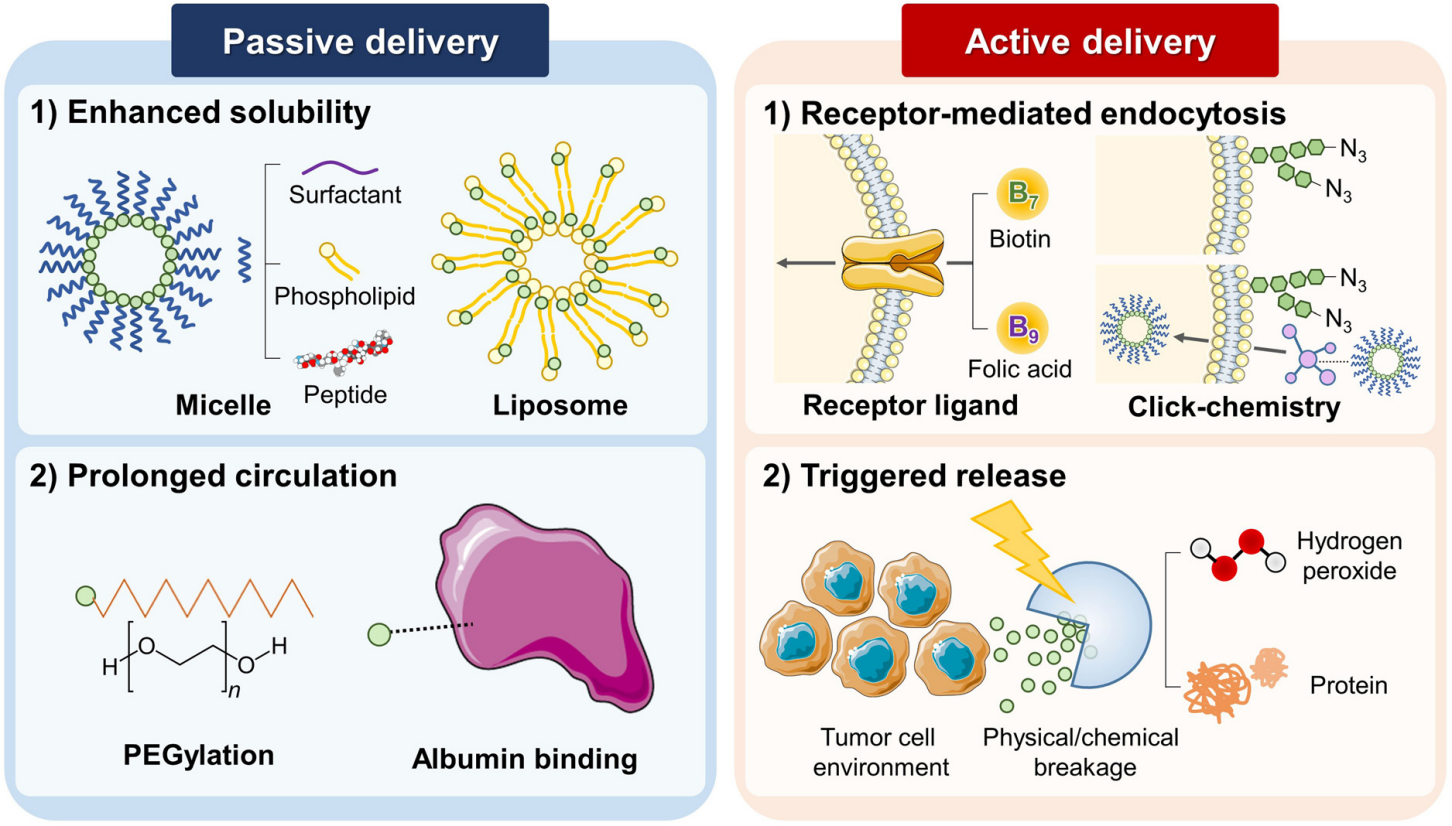
Functionalizing phthalocyanine/naphthalocyanine nanoparticles for passive and active delivery.

Low water solubility is a frequently mentioned demerit for Pc and Nc nanoparticles for using them as a PA contrast agent. The administered pharmaceutics dissolve in body fluids, e.g., blood, and get delivered to the destination tissue through the circulatory system's capillary network. Therefore, for diagnostic/therapeutic application, low water solubility is a big hurdle for tissue permeability and poses threats to stricture or stenosis. For enhancing the solubility, hydrophobic molecules are mostly coated with hydrophilic functional substances or reprocessed into amphiphilic nanocarriers with biologically suitable surfactants [Fig. 2(a)].^82,83^

When the molecular complex unit is composed of the hydrophobic part and hydrophilic part, it forms a spherical supramolecular assembly called micelle when dissolved into water. The polarity of the solvent and intermolecular interaction force grants “packing behavior,” arranging hydrophilic parts of the monomer to face the exterior surface of the assembly, and hydrophobic parts cluster toward the center of the assembly.^84–86^ The aggregates form a colloidal suspension, which leads to a better solubility of the assembly. Pluronic F127, also known as poloxamer 407, is a hydrophilic nonionic block copolymer surfactant. In pharmaceutics, pluronic copolymer micelles are commonly used as a passive drug container to improve the delivery of the hydrophobic drug.^87^ To enhance the stability of the resulting multifunctional nanodroplets, Choi *et al.* conjugated their Nc particles with the in particular, developed form of Pluronic F127 and crosslinked with the primary amine of six-arm PEG.^70,88^ By similar means, to create a lipophilic or amphipathic PC-carrying micelle, Rizvi *et al.* applied tomamine, another type of commercial surfactant. They identified an induced Pc distortion, which enabled photothermal processes for PA imaging and uncomplicated surface-enhanced resonance Raman spectroscopy.^89^ To accommodate higher loading of Nc/Pc, Zhang *et al.* introduced novel low-temperature processing called “surfactant-stripped frozen micelle.” Through the removal of free or unbound solubilizing surfactant, this processing increased the drug to surfactant ratio by 2–3 orders of magnitude higher than the conventional method.^90^ Zhang *et al.* named their surfactant-stripped high loading Nc micelles as “nanonaps” and introduced its use for multifunctional cancer theranostics.^91^

Phospholipids are the representative nature-derived amphiphilic surfactant, having hydrophilic phosphate head and two hydrophobic long fatty acid tails. The Pc/Nc enclosed with a monolayer of phospholipid forms phospholipid micelle, and the assembly is reinforced with PEG chains. Wei *et al.* reported the effective diagnostic nano agents for PA imaging, PTT, and PDT combination therapy.^58^

The liposome is another variation of a phospholipid-complex vesicle. Contrary to phospholipid micelles, liposomes consist of a lipid bilayer membrane, which can be loaded with hydrophilic nutrients or pharmaceutical drugs in its most inner void.^94^ Using such an approach, Ma *et al.* complexed ZnPc with soybean phospholipid using hydrogen bond and intermolecular interaction. The self-assembled liposomal nanoparticles were loaded with doxorubicin to achieve additional chemotherapy.^92^

Conjugating short hydrophilic peptide onto Pc/Nc particle also increases the water solubility by inducing the supramolecular self-assembly. Furthermore, short peptides may increase cellular uptake during their interaction with cell membranes due to their excellent biocompatibility.^95^ Li *et al.* suggested spatiotemporally coupled tumor phototheranostic nanoparticles, composed of easy Pc-peptide conjugation and switchable photoactivity, triggered by cellular membrane interaction.^93^

The representative publications for nanocarriers enhancing aqueous solubility are summarized in Table III.

**TABLE III.** Summary of the representative publications for nanocarriers enhancing aqueous solubility of phthalocyanine/naphthalocyanine nanoparticles. $\bar{d}$, mean diameter; PEG, polyethylene glycol, $\lambda_{\text{max}}$, maximum absorption wavelength; ϵ, molar extinction coefficient; $\Phi_{F}$, fluorescence quantum yield; $\Phi_{\Delta }$, singlet oxygen generation quantum yield; SERS, Surface-enhanced Raman spectroscopy; η, photothermal conversion coefficient; PDT, photodynamic therapy; PTT, photothermal therapy.

Group	Nanomaterial	Carrier type	Excipient type	$\bar{d}$ (nm)	Major features
Choi *et al.*^70^	Nc/PFH@PCPN	Block copolymer micelle	Six-arm PEG-amine crosslinked Pluronic F127	500–2500	• $\lambda_{\text{max}}$ = 850 nm
					• 89.4 % 14-day sustainability (37 °C)
Rizvi *et al.*^89^	ZnPc (tomamine)_n_	Block copolymer micelle	Tomamine	89 ± 8;	• $\lambda_{\text{max}}$ [nm] = 774; 810; 805; 820
				133 ± 7;	• ϵ [O.D.] = 3.60; 3.49; 3.38; 3.25
				193 ± 10;	• $\Phi_{F}$ ≪ 1
				223 ± 9;	• $\Phi_{\Delta }$< 0.01
					• strong SERS signal
Zhang *et al.*^91^	Nanonap	Block copolymer micelle	Pluronic F127	29.5	• Surfactant-stripped micelle
					• $\lambda_{\text{max}}$ = 860 nm
					• PTT with upconversion cream,
					• PET application with ^64^Cu chelation
Wei *et al.*^58^	SiNcOH-DSPE-PEG(NH_2_)	phospho-lipid micelle	PEG-conjugated phospholipid	160	• η = 59.8 %
					• 7-day stability
Ma *et al.*^92^	ZnPc-SPC	Liposome	ZnPc-soybean phospholipid complex	296.9 ± 6.553	• Drug loading (doxorubicin)
					• Folate receptor(FRα),
					• max. 15 day stability
Li *et al.*^93^	Pc-petide conjugate self-assembly	Short Petide micelle	L-phenylalanine-L-phenylalanine	54.8 ± 17.6 (pH 7.0)	• Theranostic application (PDT/PTT)
					• pH dependent nanoparticle size
					• Switchable photoactivity with cell membrane interaction

#### 2. Prolonged circulation

The capillary network delivers the injected nanoparticles into cells via the reticuloendothelial system using the principle of diffusion. The difference between hydraulic pressure inside the capillary wall and intracellular pressure of the recipient cell transfers oxygen and nutrients essential for metabolic events of the cell. The enhanced permeability and retention effect (EPR) is a generally accepted phenomenon in vessels surrounding rapidly growing tumor cells. These tumor cells have greater cellular uptake through large fenestrations between endothelial cells and allow nanoparticles to reach the matrix easily than normal cells.^96^ Thus, the prolonged circulation of the nanoparticle increases the possibility of them reaching the peritumor site, and is necessary for efficient passive delivery of the nanoparticle.

Many methods such as cross-linking with polyethylene glycol (PEG) classes and variants, or PEGylation, are adopted to extend the circulation time of the injected nanoparticles by growing the volume of the nanocarrier, which effectively inhibits the reticuloendothelial uptake.^97^ In addition, bulky organic ligands on the monomer provides steric hindrance and lowers the critical micellar concentration, which degrades the lower intramolecular aggregation.^98^ Huang *et al.* modified an Sn-chelated octabutoxy Nc with PEG, and demonstrated increased blood circulation after intravenous administration to the mouse in *in vivo* experiment with 24 hours monitoring. The experimental results showed 4 times longer half-life and 10 times greater area under the curve.^99^

Albumin binding is also a well-preferred method to regulate the circulation time. Human serum albumin is the most abundant protein in human blood plasma; it constitutes about 50%f of serum protein and is produced in the liver. Against the hydraulic pressure that diffuses the solvents to peripheral cells during capillary metabolism, albumin regulates the oncotic pressure of blood by trapping water, cations (e.g., Ca^2+^, Na^+^, K^+^), fatty acids, hormones, bilirubin, tyroxine, thus preventing discharge of pharmaceuticals from microvessels. The passive delivery rate of drugs is often enhanced by binding with albumin in the pharmaceutical field.^100^ Using inherent biocompatibility and reduced immunogenicity, Jia *et al.* fabricated a human serum albumin-iron(II) Pc nanoparticle complex as a phototheranostic agent and observed the monotonic increase in PA signal intensity at < 15 hours post-injection.^101^

The representative publications for absorption spectrum shift are summarized in Table IV.

**TABLE IV.** Summary of the representative publications for prolonged circulation of phthalocyanine/naphthalocyanine nanoparticles. PA, photoacoustic; $\lambda_{\text{max}}$, maximum absorption wavelength; AUC, area under the curve; η, photothermal conversion coefficient; $\bar{d}$, mean diameter; ζ, zeta potential.

Group	Nanomaterial	Ligand type	Max. PA signal elapse (H)	Half lifetime (h)	Mean residence time (h)	Major features
Huang *et al.*^99^	PEG-Sn-ONc	PEG	24	5.6 ± 1.2	8.1 ± 1.8	• $\lambda_{\text{max}}$ = 930 nm
						• 4 times longer half-life
						• ∼10 times AUC.
Jia *et al.*^101^	HSA-FePc NPs	Human serum albumin	12	⋯	⋯	• η = ∼ 44.4% (at 671 nm)
						• $\bar{d}$ = 55 nm
						• ζ = −26.9 mV
						• Low long-term toxicity
						• Preferential accumulation to tumor site
						• ∼51.3 wt% loading efficiency

### C. Active delivery

In addition to the passive delivery, engineering the nanoparticles with targeting or stimulus-triggered release function helps to achieve optimal therapeutic and diagnostic effects [Fig. 2(b)].^102^ The presence of closed d-shell diamagnetic ions (e.g., Zn^2+^, Al^2+^, Ga^3+^) strengthens Pc/Nc complexes of both high triplet yields and long lifetimes and consequently forms hydroperoxide products (type I), or yield singlet oxygen generation converted from ground-state triplet oxygen (type II).^67^ Both oxidative reactions degrade cellular components, and the damage level highly depends on the local concentration of the dye and oxygen availability. Excessive dosage into the living matter may adversely occur damage to the nontarget systemic normal cells together with targeting tumor cells and lead to pathologic response.^103,104^ Therefore, selective Pc/Nc delivery restricted only to the targeting tumor cells becomes important to minimize the dosage needed for the treatment. The designed nanocarriers prevent the excessive release of dosage into the bloodstream and normal tissues and therefore keep up the delivery to desired tumor tissue, resulting in high drug concentration within the tumor. From this section, we explain both concepts into the boundary of “active delivery,” as the technique selectively marks the target tumor cells depending on nanoparticle-cell interaction under specific target cell conditions.^105^ The representative publications for absorption spectrum shift are summarized in Table V.

**TABLE V.** Summary of the representative publications for active delivery of phthalocyanine/naphthalocyanine nanoparticles. η, photothermal conversion coefficient; $\bar{d}$, mean diameter; PA, photoacoustic, FL, fluorescence, PDT, photodynamic therapy.

Group	Material name	Delivery type	Mechanism	Major features
Wu *et al.*^110^	Biotin-conjugated ZnPc nanodots	Receptor-mediated endocytosis	Biotin receptor	• η = 45.7%
				• $\bar{d}$ = 80 nm
				• Max. PA signal in +8 hr post-injection
				• Increased phototoxicity with receptor-rich HeLa cell
Ding *et al.*^57^	PEG-folate/ZnPc nanodots	Receptor-mediated endocytosis	Folic acid receptor	• $\bar{d}$ = 20 nm
				• Effectively internalized to folate receptor-overexpressed CNE-2 cell
Du *et al.*^113^	DBCO-ZnPc-LP	Receptor-mediated endocytosis	Bioorthogonal Metabolic Glycoengineering-Activated Tumor Targeting	• $\bar{d}$ = 78.82 nm
				• Complete self-quenching
				• η = ∼ 44.39%
Xie *et al.*^115^	PCBP	Triggered release	H2O2-triggered self-immolative PEG arms	• $\bar{d}$ = max. ∼350 nm
				• ∼1.4-fold PA amplitude increase
Li *et al.*^116^	NanoPcTB	Triggered release	Photoswitchable protein disassembly	• Biotin receptor-mediated switchable property (FL/PDT)
Li *et al.*^93^	Pc-peptide conjugate self-assembly	Phthalocyanine-peptide conjugate self-assembly	Photoswitchable protein disassembly	• L-phenylalanine-L-phenylalanine (FF)—mediated switchable photoactivity (FL/PDT)

#### 1. Receptor-mediated endocytosis

In active targeting, ligands bonded to the nanoparticle promote receptor-mediated endocytosis specifically to the receptor-acquainted cells. The nanoparticles engineered with active delivery are often multi functionalized not only for the diagnostic imaging purpose but also for the therapeutic purpose. The binding biomolecule, such as biotin (or vitamin B7) or folic acid (or vitamin H), which combines overexpressed receptors on the cell membrane, is considered a promising biomarker for substantially reducing the streak effects of aggressive pharmaceutics.^106^

Studies have revealed the tendency of biotin receptors to overexpress in tumors than in normal tissues. Besides, the structure of biotin is simple and can be readily functionalized.^107–109^ Thus, it has the potential to be an active tumor-targeting ligand. Wu *et al.* produced a tumor-targeting capability of fabricated ZnPc nanodots by conjugating biotin onto the surface.^110^ Also, Ding *et al.* equipped tumor-specific delivering property by targeting ligand by coupling ZnPc nanodots with folic acid-conjugated PEG chains.^57^

To overcome the intrinsic disadvantages of traditional biological molecules, such as a limited number of receptors and tumor heterogeneity, a two-step chemical tumor-targeting concept, composed of metabolic glycoengineering and click chemistry, has been actively applied from the time it was first proposed by Koo *et al.*^111,112^ The addition of artificial chemical receptor increases the number of binding nanocarriers significantly on the surface of most tumor cells, regardless of tumor type. Such a strategy was first presented by Du *et al.* in the photothermal/photoacoustic synergistic therapy application.^113^

#### 2. Triggered release

The difference between receptor-mediated endocytosis and triggered release is that the latter involves a physical or chemical breakage of nanocarrier structure and is triggered by a specific cell condition. Hydrogen peroxide or H_2_O_2_-triggered drug release is designed for exclusive oxidative microenvironment within cancer cells.^114^ The ROS responsive nanocarrier rapidly disassembles in target cells and promotes rapid intracellular drug release. The four self-immolative PEG arms consisting of the activatable probe, developed by Xie *et al.*, are specifically cleaved with the ROS presence. The increased hydrophobicity promotes the supramolecular regrowth around the tumor cells and gets spatiotemporally highlighted in the photoacoustic image.^115^

Joining selective recognition of protein as a biomarker and reverse application of self-quenching monomer could lead to the novel design of switchable photoactivity. Li *et al.* explored biotin-receptor-responsive photoswitchable property from biotin-conjugated Pc-based nanodots.^116^ The presence of biotin-receptor-like protein triggers the nanoparticle's partial disassembly, resulting in the generation of fluorescence and reactive oxygen species instead of photothermal conversion, inducing PA signal generation and PTT effect. The nanoparticle delivery showed high selectivity toward biotin receptor-positive cancer cells on xenograft models (A549), compared to the receptor-negative cancer cells (WI38-VA13). The switchable photoactivity from short peptide micelle was also reported in aforementioned Li *et al.*'s work.^93^

## III. BIOMEDICAL APPLICATIONS OF P_c_/N_cs_

### A. Biomedical Imaging

#### 1. Contrast-enhanced PA imaging

Using endogenous biological chromophores, such as oxy-/deoxy-hemoglobin and melanin, the PA imaging achieves superb optical imaging contrast, high spatial resolution, and a larger depth of penetration compared to pure optical imaging techniques. However, due to strong light absorption and scattering in tissue, light fluence and signal-to-noise ratio (SNR) decrease exponentially with increased imaging depth. In addition, some tissues or organs that need to be imaged, such as tumor and lymphatic system, do not possess intrinsic optical contrast. Many Pc/Nc-based materials have been developed as PA imaging contrast agents to visualize such biological targets and improve imaging sensitivity, specificity, and penetration depth.^78,99,117–119^

**FIG. 3.**
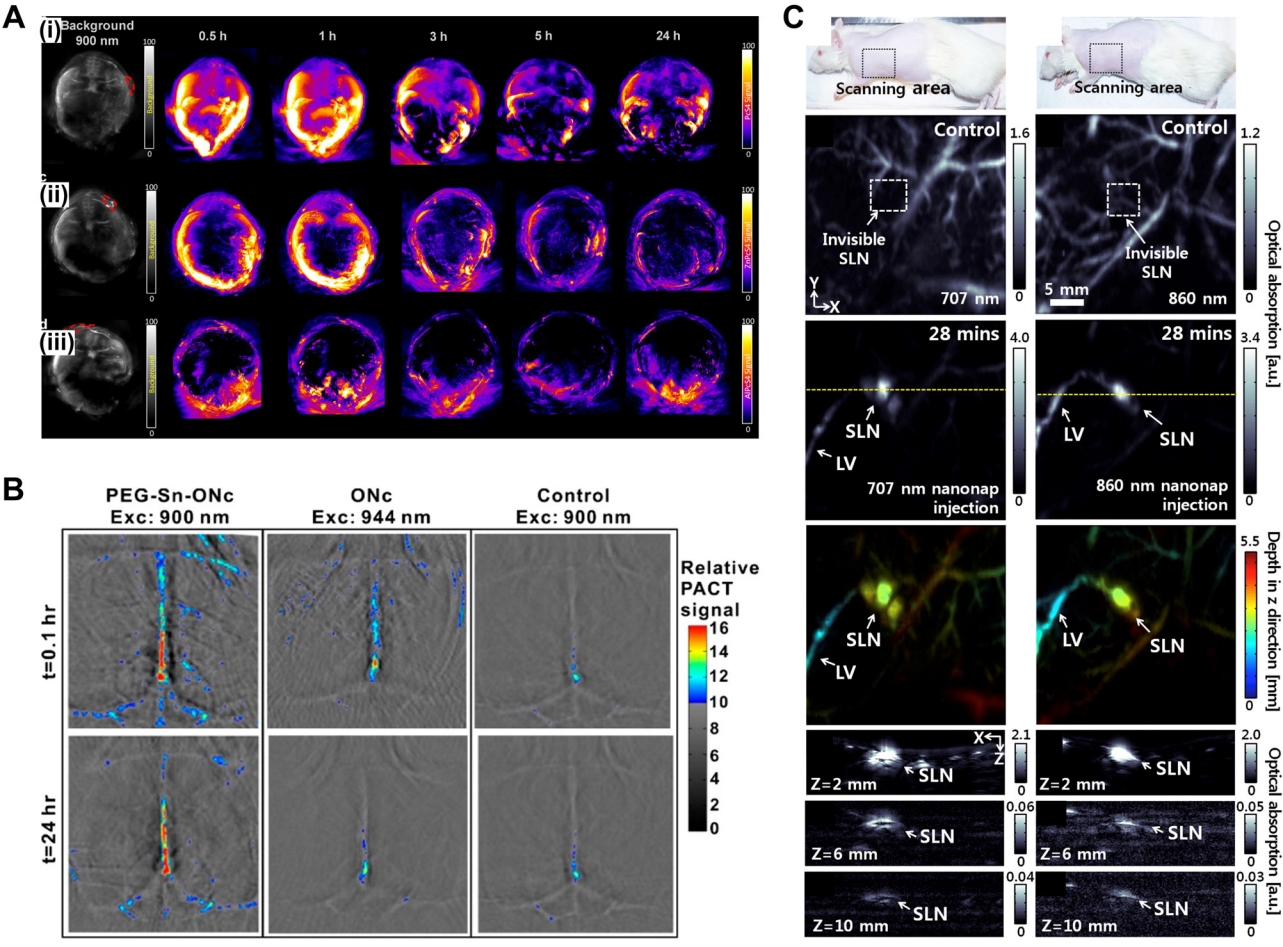
Contrast-enhanced PA images. (a) *In vivo* PA signals at various time points after tail-vein administration of (i) PcS4, (ii) ZnPcS4, and (iii) AlPcS4. Reprinted with permission from Attia *et al.*, Biomed. Opt. Express **6**(2), 591 (2015). Copyright 2015 The Optical Society. (b) Noninvasive photoacoustic images of brain blood vessels of mice administered with PEG-Sn-ONc or ONc at the indicated time points following intravenous injection. Reprinted with permission from Huang *et al.*, Bioconjugate Chem. **27**(7), **1574** (2016). Copyright 2016 American Chemical Society. (C) *In vivo* PA imaging of rat's SLNs with injection of 707 nm (left column) and 860 nm (right column) nanonaps. Reprinted with permission from Lee *et al.*, “Dual-color photoacoustic lymph node imaging using nanoformulated naphthalocyanines,” Biomaterials **73**, 142–148. Copyright 2015 Elsevier. PA, photoacoustic; SLN, sentinel lymph node; LV, lymphatic vessel.

For *in vivo* PA tumor imaging, Attia *et al.* investigated three tetrasulfonate phthalocyanine formulations as contrast agents: phthalocyanine tetrasulfonic acid (PcS4), Zn(II) phthalocyanine tetrasulfonic acid (ZnPcS4), and Al(III) phthalocyanine chloride tetrasulfonic acid (AlPcS4).^118^ They observed high PA imaging contrast in the tumor after intravenous administration of PcS4 [Fig. 3(a)]. Huang *et al.* synthesized Sn(IV)-chelated octabutoxy Nc (Sn-ONc) with axial conjugation of polyethylene glycol (PEG) for photoacoustic vascular imaging [Fig. 3(b)].^99^ Introducing Sn and PEG exhibited longer absorption peaks and prolonged circulation time, respectively, enabling noninvasive long-term brain vessel imaging *in vivo*. A family of organic nano formulated naphthalocyanine was first introduced by Zhang *et al.*^119^ The reported nanoparticle was designed using the formulation of frozen micelles to endure a hostile gastrointestinal (GI) environment for real-time PA imaging of GI function. Based on the tunable NIR absorption wavelength of the nanoformulated Nc, Lee *et al.* developed two nanoformulated Nc dyes with large NIR absorption at 707 nm or 860 nm using 2,11,20,29,tetra-tert-butyl-2,3-naphthlaocyanine or 5,9,14,18,23,27,32,36-octabutoxy,-2,3-naphthalocyanine, respectively. They photoacoustically observed lymphatic vessels and the sentinel lymph node [Fig. 3(c)], which are invisible in label-free PA imaging.^117^

#### 2. Multimodal imaging agents

Thanks to the inherent capability of Pc/Nc for high NIR absorption and centric metal ion chelation, Pc/Ncs have great potential to be a contrast agent for complementary multimodal imaging.

The FL is one of the most widely used biological imaging modalities in visualizing living organelles. When a substance absorbs light, the excess energy dissipates through the emission of light, which is FL and/or heat, contributing to PA wave generation. Though FL and PA have competing yield, both are based on high absorbance of light, and thus, many contrast agents have been reported as a bimodal FL and PA imaging agent or FL-PA switchable imaging agent.^68,81,92,93,116,120–122^ Li *et al.* developed phthalocyanine-peptide conjugate nanoparticles with switchable photoactivity between PA imaging, associated with photothermal therapy, and FL imaging, associated with photodynamic therapy.^93^ The switchable photoactivity was observed when the NPs were disassembled by the cell membrane-interaction, which permitted spatiotemporally optimal therapeutic windows via adaptive PA/FL imaging, and achieved precise phototheranostics.

With a nature of PA imaging of combining photon excitation and US detection, the PA imaging can be easily integrated with the conventional US imaging. Choi *et al.* designed surface crosslinked nanodroplets encapsulating Nc and perfluorohexane for PA/US dual-modal image-guided high-intensity focused ultrasound (HIFU) therapy.^70^ The accumulation of the developed nanoparticles in the tissue was photoacoustically monitored to confirm the optimal time point of HIFU treatment. The intratherapy monitoring was performed by US imaging visualizing echogenic microbubbles generated under HIFU exposure.

**FIG. 4.**
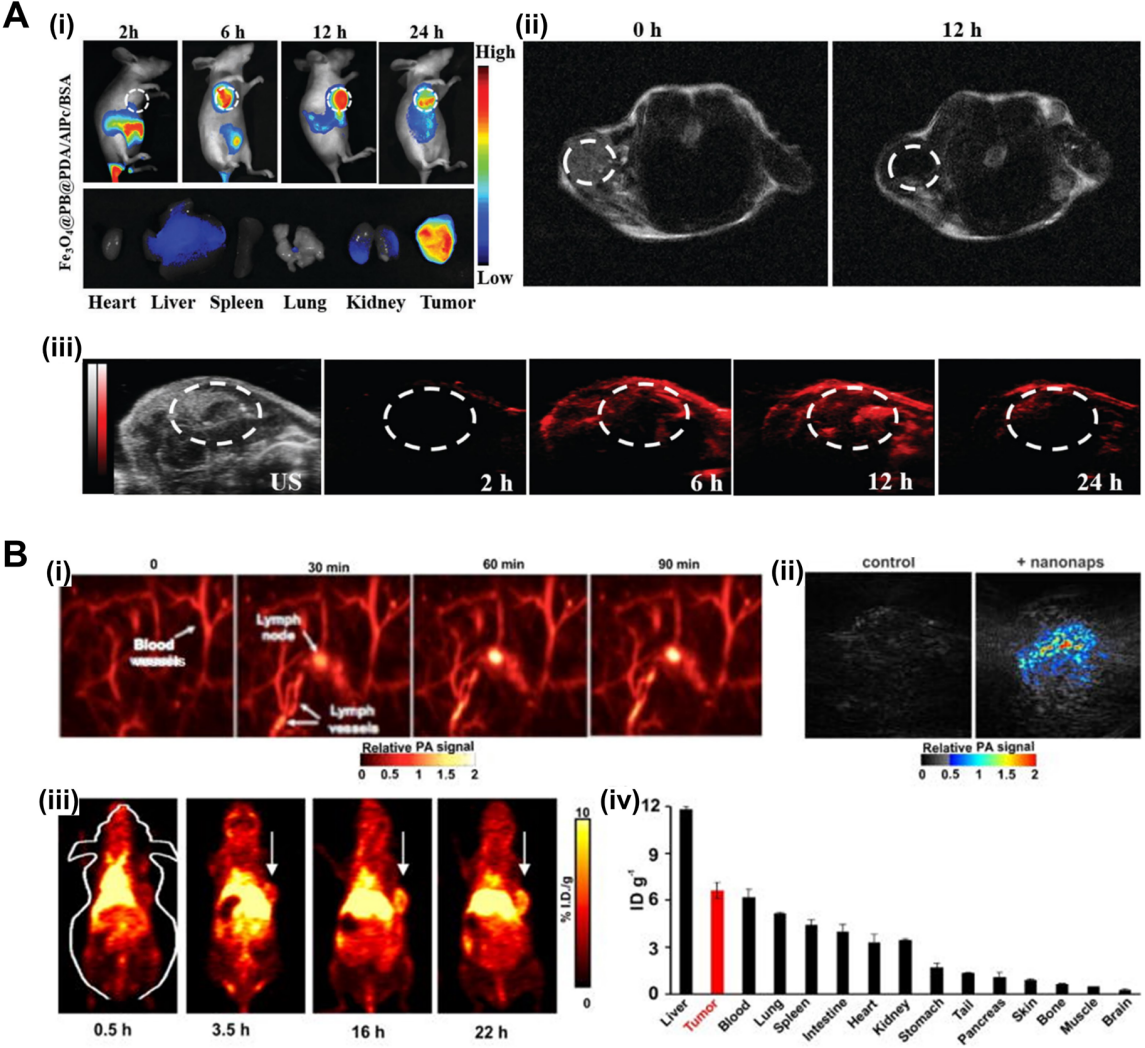
Pc/Ncs for multimodal imaging. (a) (i) *In vivo* FL images after i.v. injection of Fe_3_O_4_@PB@PDA/AlPc/BSA in tumor-bearing mice at 2 h, 6 h, 12 h, 24 h and *ex vivo* FL images of AlPc in major organs induced with 660 nm laser irradiation after i.v. injection for 24 h. (ii) MR signals in the tumor before and 12 h after i.v. injection with Fe_3_O_4_@PB@PDA/AlPc/BSA. (iii) US and PA images of tumor-bearing mice after i.v. injection with Fe_3_O_4_@PB@PDA/AlPc/BSA taken different time points. Republished with permission from Wang *et al.*, J. Mater. Chem. B **6**(16), 2460 (2018); permission conveyed through Copyright 2018 Royal Society of Chemistry, Clearance Center, Inc. (B) Nanonaps for PA and PET imaging. (i) PA lymph node imaging using nanonaps. (ii) PA imaging of subcutaneous 4T1 whole tumors in living BALB/c mice with or without i.v. administration of 2.6 mg nanonaps 24 h prior. (iii) Serial PET images of 4T1 subcutaneous breast tumors in BALB/c mice after i.v. injection. Arrows show tumor location. (iv) Biodistribution of ^64^Cu within nanonaps 24 hours post injection of nanonaps. Republished with permission from Zhang *et al.*, Nanoscale **9**(10), 3391 (2017); permission conveyed through Copyright 2017 Royal Society of Chemistry Clearance Center, Inc. FL, fluorescence; i.v., intravenous; MR, magnetic resonance; US, ultrasound; PA, photoacoustic; PET, positron emission tomography.

Other imaging modalities have also been explored using Pc/Nc-based PA agents. Wang *et al.* fabricated polydopamine (PDA)/aluminum Pc (AlPc)/bovine serum albumin (BSA) coated magnetic Prussian blue nanoparticles for triple-modal FL/MR/PA cancer imaging [Fig. 4(a)].^120^ Lu *et al.* compared and screened a series of porphyrin Pc and Nc dyes, encapsulated in the interior of nanoparticles through the flash nanoprecipitation (FNP) process, and revealed strong PA responses at high loadings of dyes and combined PA and FL responses at lower loadings of dyes.^68^ The incorporability of nanoparticles with MR/PET/SPEC imaging agents and therapeutic drugs was also demonstrated. Zhang *et al.* developed nanoformulated Ncs for *in vivo* dual-modal PA/PET imaging of lymph nodes and tumors [Fig. 4(b)].^91^

The representative publications for biomedical imaging application are summarized in Table VI.

**TABLE VI.** Summary of the representative publications for biomedical imaging application of phthalocyanine/naphthalocyanine nanoparticles. $\lambda_{\mathrm{ex}}$, excitation wavelength; PA, photoacoustic; GI, gastrointestinal tract; NP, nanoparticle; FL, fluorescence; PTT, photothermal therapy; PDT, photodynamic therapy; US, ultrasound; HIFU, high-intensity focused ultrasound; MR, magnetic resonance; PET, positron emission tomography.

Group	Agent	$\lambda_{ex}$ (nm)	Imaging modality	Application	Major features
Huang *et al.*^99^	PEG-Sn-ONc	900, 944	PA	Blood circulation (brain)	• Absorbance shift from 860 nm to 930 nm via Tin chelation
					• Prolonged circulation time via axial PEGylation
Zhou *et al.*^78^	P-Pc	1064	PA	Tumor imaging, GI tract	• High absorbance (>1000) at 1000 nm
					• Imaging depth: 11.6 cm (chicken breast tissues), 5 cm (human arm)
Lee *et al.*^117^	2,11,20,29,tetra-tert-butyl-2,3-Nc or 5,9,14,18,23,27,32,36-octabutoxy,-2,3-Nc	707, 860	PA	Lymphatic system imaging	• Dual-color PA imaging of two separate lymphatic systems
					• Spectrally stable at a very high concentration
Li *et al.*^116^	PF NP	680	PA/FL	Bimodal image-guided PTT/PDT	• Switchable PTT+PA and PDT+FL theranostics
					• Highly selective for biotin receptor-positive cancer cells (e.g. A549)
Choi *et al.*^70^	Nc/PFH@PCPN	850	PA/US	Bimodal image-guide HIFU therapy	• 0 US imaging contrast when vaporized under HIFU ablation
Wang *et al.*^120^	Fe_3_O_4_@PB @PDA/AlPc/BSA	660	PA/FL/MR	Trimodal image-guided PTT/PDT	• Fe_3_O_4_-based MR contrast
					• Prussian blue (PB) modification for PA-guided PTT
					• Synergistic FL-guided PDT with AlPc photosensitizer
Zhang *et al.*^91^	Surfactant-stripped ONc nanonap	860	PA/PET	Lymphatic mapping, tumor imaging	• ^64^Cu-based PET contrast

### B. Probing biological processes

#### 1. Tumor detection

**FIG. 5.**
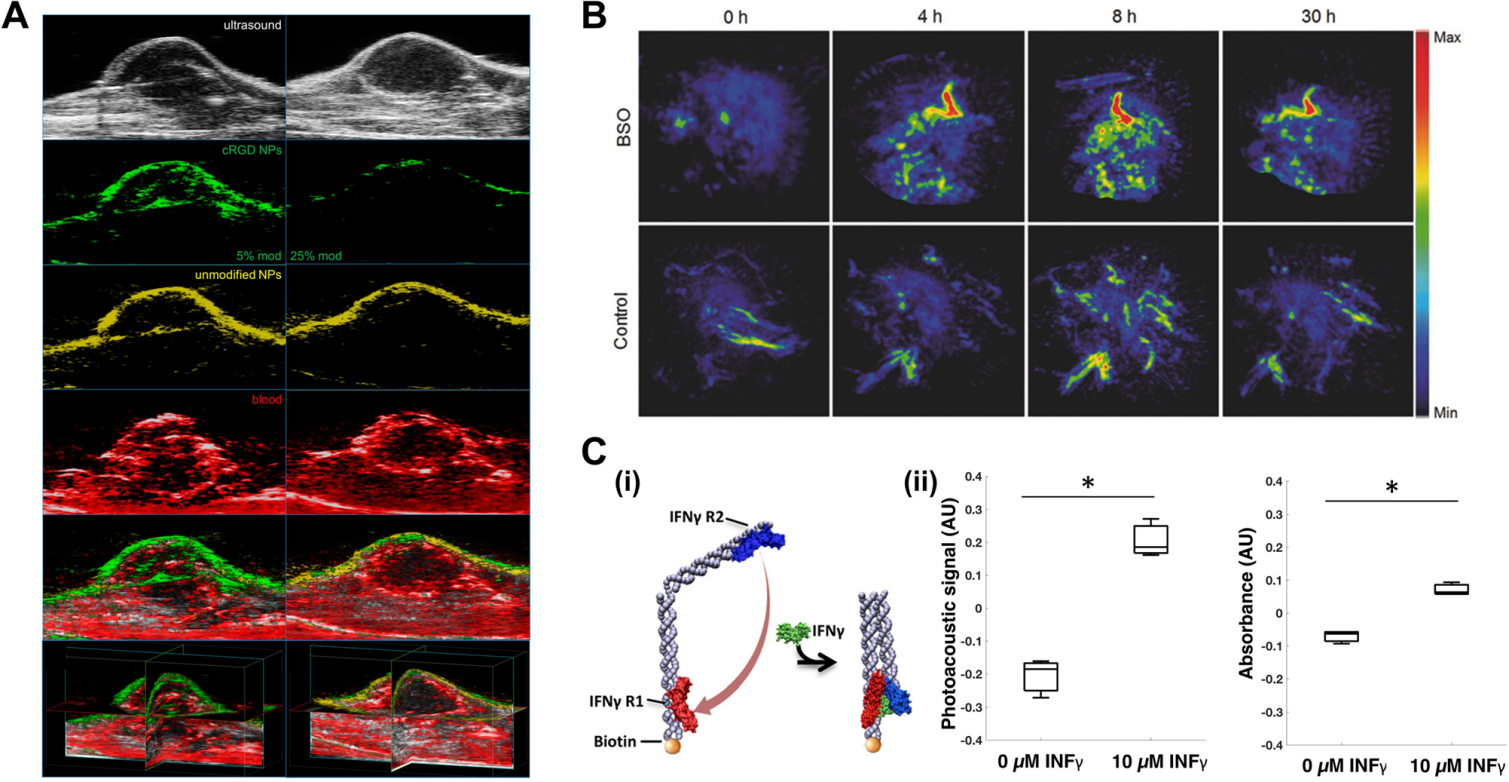
Pc/Nc-based agents for probing biological processes. (a) Multiplexed *in vivo* tumor PA imaging. Representative 2D slice images exhibiting US (first row), cRGD NP PA (second row), unmodified NP PA (third row), blood PA (fourth row), and overlaid signals (fifth row), and example 3D reconstruction (sixth row) of mice tumors, 48 h after tail vein inoculation with 5% cRGD NPs + 0% cRGD NPs (left column) and 25% cRGD NPs + 0% cRGD NPs (right column), respectively. Reprinted with permission from Lu *et al.*, ACS Biomater. Sci. Eng. **3**(3), 443 (2017). Copyright 2017 American Chemical Society. (b) Representative PA MAP images of tumors for BSO-pretreated and untreated mice after systemic administration of PCBP (30 μg per mouse) through tail vein. Republished with permission from Xie *et al.*, Adv. Mater. **29**(44), 1703693 (2017). Permission conveyed through Copyright 2017 John Wiley and Sons Clearance Center, Inc. (C) (i) Schematic of the nanosensor 2-step binding mechanism between IFNγ and its receptors. (ii) Response of the IFNγ nanosensor (780 nm) to buffer and 10 *μ*M IFNγ (169 *μ*g/ml) in buffer. Error bars represent the standard deviation of three independent trials. Reprinted with permission from Morales *et al.*, ACS Sens. **4**(5), 1313 (2019). Copyright 2019 American Chemical Society. PA, photoacoustic; US, ultrasound.

Active targeting for anticancer is essential for delivering theragnostic agents to the region of interest while avoiding normal tissues. Several Pc/Nc derivatives have been introduced as an active cancer diagnostic probe or to deconvolute passive vs active targeting effects. As an actively targeted cancer theragnostic agent, Ma *et al.* presented a zinc phthalocyanine soybean phospholipid (ZnPc-SPC),^92^ which showed high sensitivity to FRα over-expressed tumor cells. The ZnPc-SPC complex presented multiphase theranostics: (1) PTT associated with PA imaging in the early phase and (2) PDT with FL images in the late phase accompanied by pH-sensitive drug release in the tumor. Lu *et al.* developed multiplexed PA imaging agents of cRGD-modified nanoparticles, which can bind to α_V_β_3_ integrin expressed in Lewis lung carcinoma tumors, and unmodified nanoparticles based on Pc/Nc macrocycles, which can internally normalize active ligand targeting effects from passive targeting such as EPR effect.^123^ PA imaging of the two spectrally separable nanoparticles showed the distinct distribution of agents in the tumor site [Fig. 5(a)]. Du *et al.* investigated tumor-targeting Pc compounded with dual-targeting effect via metabolic glycoengineering and click chemistry.^113^ The PA signal in the tumor region was substantially higher in the mice group injected with the proposed dual-targeting nanoagent intravenously than in the control group with a passive-targeting agent.

#### 2. Biomolecule detection

Tracking biomolecules in the body is critically important in many fields ranging from basic science research to medical health care. The tracking helps significantly in understanding biological/physiological processes in living organisms, diagnosing diseases, or monitoring pre-/in-/post-treatment responses. Recently, Morales *et al.* developed a DNA-based PA nanosensor to detect interferon-gamma (IFNγ) that plays a critical role in activating immunity against infections.^124^ In the presence of IFNγ, the DNA structure's arms were folded to induce stacking of Pc dye, achieving a 55% increase in PA signal [Fig. 5(c)]. Toriumi *et al.* reported tautomeric benziphthalocyanines, by introducing free hydroxyl group with esterase- or H_2_O_2_-labile markers, for activatable PA detection of esterase or H_2_O_2_.^55^ Xie *et al.* designed Pc-based semiconducting macromolecule, which first underwent ROS-induced cleavage process of PEG and then subsequently the residual Pcs self-assembled into large nanoparticles, showing enhanced PA signal [Fig. 5(b)] in the presence of ROS.^115^

The representative publications for probing biological processes with Pc/Ncs are summarized in Table VII.

**TABLE VII.** Summary of the representative publications for probing biological processes with phthalocyanine/naphthalocyanine nanoparticles. PTT, photothermal therapy; PA, photoacoustic; PDT, photodynamic therapy; FL, fluorescence; ROS, reactive oxygen species.

Group	Agent	Targeting/Probing strategy	Application
Ma *et al.*^92^	ZnPc-SPC	FRα receptor	• FRα over-expressed tumor cell detection (e.g. 4T1)
			• Multiphase phototheranostics (Phase I: PTT/PA → Phase II: PDT/FL)
			• pH-sensitive drug release in tumor
Lu *et al.*^123^	PEG-coated Pc/Nc with *tert*-butyl or butoxy functional groups with/without cRGD surface modification	α_V_β_3_ integrin	• Multiplexed PA imaging
			• Lewis lung carcinoma tumor detection
Du *et al.*^113^	DBCO-ZnPc-LP	Metabolic glycoengineering + click chemistry	• PA tumor imaging
			• PA image-guided PTT/PAT
Morales *et al.*^124^	DNA-based nanostructure conjugated with Pc and IFNγ receptors	IFNγ receptors (IFNγR1, IFNγR2)	• IFNγ detection
Toriumi *et al.*^55^	BPcs derivatives	Free hydroxyl group with esterase-/H_2_O_2_-labile markers	• Activatable PA detection of esterase or H_2_O_2_
Xie *et al.*^115^	Macromoleuclar PCBP	Four PEG arms with ROS-responsive linker, inducing self-assembling upon ROS-induced cleavage of PEG	• PA detection of ROS during drug treatment

### C. Therapeutic agents

#### 1. Photothermal therapy

**FIG. 6.**
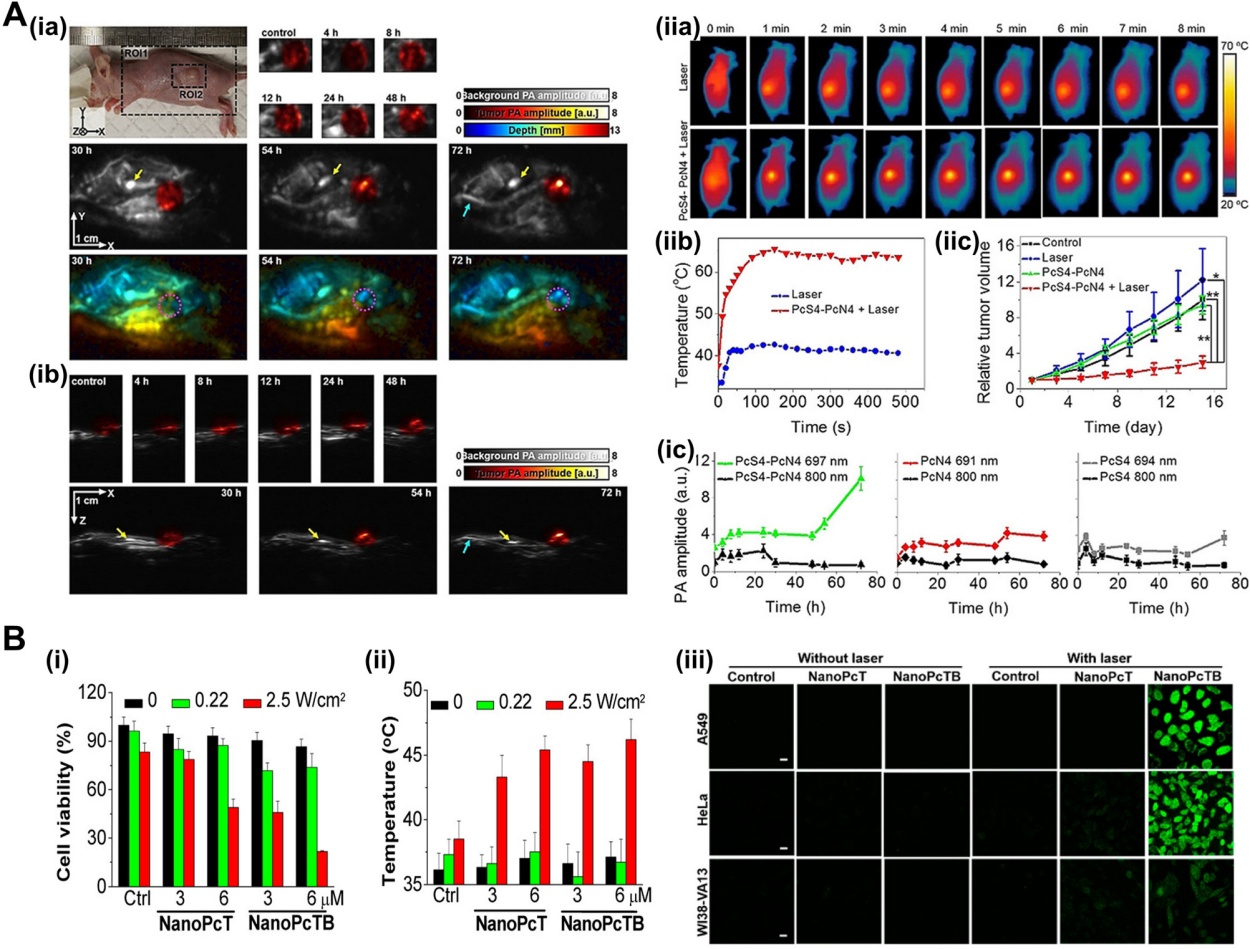
Pc/Nc-based agents for PA image-guided phototherapy. (a) *In vivo* PA (ia) top-view and (ib) side-view MAP images of 4T1-bearing mice before and after intraperitoneal injection of PcS4-PcN4. The gray-scale image represents background PA amplitude, color-scale images represent PA amplitude in the tumor region and depth along the z axis. (ic) *In vivo* PA amplitude changes in the tumor region over time before and after the injection of PcS4, PcN4, and PcS4-PcN4. (iia) Thermal IR imaging of 4T1-bearing mice with the indicated treatments. (iib) Temperature change curves of 4T1 tumors in mice after the indicated treatments. (iic) Growth curves of tumors in mice after the indicated treatments. Republished with permission, from Li *et al.*, Angew. Chem. **59**(22), 8630–8634 (2020). Copyright 2020 John Wiley and Sons Clearance Center, Inc. (b) *In vitro* anticancer effect. (i) Cytotoxic effects and (ii) temperature of the HeLa cells incubated with NanoPcT and NanoPcTB for 2 h without or with 655 nm laser irradiation. (iii) ROS generation induced by NanoPcT and NanoPcTB in the A549, HeLa, and WI38-VA13 cells in the presence of laser irradiation. Scale bar = 20 *μ*m. Reprinted with permission from Li *et al.*, J. Am. Chem. Soc. **139**(31), 10880 (2017). Copyright 2017 American Chemical Society. PA, photoacoustic; MAP, maximum amplitude projection; ROS, reactive oxygen species.

Since PA imaging is based on high optical absorption and local heat conversion, many Pc/Ncs investigated for PA imaging agents inherently have great potential for PTT, which is based on local heat generation at a specific excitation band. The Pc/Ncs, mainly due to strong absorbance at long wavelengths, possess attractive traits of theranostics such as deeper penetrability, lower photon energy, and much safer to adjacent normal cells/tissues. In 2020, Li *et al.* reported a nanostructured theranostic agent for both PA imaging and PTT through supramolecular self-assembly of two water-soluble Pc derivatives.^54^ The Pc assemblies showed completely quenched FL and strong PA/photothermal responses, resulting in high contrast PA imaging of cancer tumors and efficient anticancer PTT *in vivo* [Fig. 6(a)]. To further exploit the advantages of maximum permissible exposure (MPE) of laser and deep penetration in tissues, a phototheranostic agent in the second near-infrared (NIR-II) window was proposed by Pan *et al.*^56^ The agent was developed based on an organic molecular material, cruciform Pc pentad. The *in vitro* and *in vivo* studies demonstrated strong PA signals and PTT effect at 1064 nm. Du *et al.* developed a tumor-targeting nanoagent, dibenzyl cyclooctyne zinc(II)-Pc lipid-poly(ethylene glycol) (DBCO-ZnPc-LP), for multifunctional phototheranostics of PTT and PA therapy, which is a recently emerged phototherapy to eradicate tumor cells using PA shockwave.^113^

#### 2. Photodynamic therapy

The Pc/Ncs have been widely exploited as photosensitizers in PDT due to strong absorption at long wavelengths and easily tunable photochemical properties. Ding *et al.* synthesized zinc(II)-phthalocyanine (ZnPc) nanodot by cryodesiccation-driven crystallization for PA image-guided cancer PDT.^57^ In addition to the PDT capability, with cytotoxic singlet oxygen generation, the nanodots showed excellent water-soluble and stealth properties with the surface modified using PEG_2000_-folate and Pluronic F127. Li *et al.* developed nanostructured Pc assemblies as a PDT agent, which are activatable in biotin receptor-positive cancer cells.^116^ The Pc assemblies displayed synergistic phototheranostic capability with intrinsic PA and PTT properties and cancer-specific FL and PDT properties [Fig. 6(b)]. The two-photon excited PDT (TPE-PDT) was introduced by Mauriello-Jimenez *et al.* for precise cancer treatment.^122^ Similar to two-photon excitation microscopy, The TPE-PDT allows deep penetration with longer wavelengths and superb spatiotemporal resolution. Bridged silsesquioxane nanoparticles were designed from tetrasilylated porphyrin and Pc, showing excellent TPE-PDT performance and strong PA imaging contrast *in vivo*.

The representative publications for phototherapy with Pc/Nc nanoparticles are summarized in Table VIII.

**TABLE VIII.** Summary of the representative publications for phototherapy with phthalocyanine/naphthalocyanine nanoparticles. PA, photoacoustic; PTT, photothermal therapy; FL, fluorescence; NIR, near infrared; PAT, photoacoustic therapy; PDT, photodynamic therapy.

Group	Agent	Phototherapy parameters	Application	Major features
Li *et al.*^54^	PcS4-PcN4	660 nm, 2 W·cm^−2^, 8 min	PA image-guided PTT	• Completely quenched FL, leading to strong PA/PTT responses
Pan *et al.*^56^	Zn_4_-H_2_Pc/DP NPs	1064 nm, 0.6 W·cm^−2^, 10 min	PA image-guided PTT	• Phototheranostic agent in NIR-II window
Du *et al.*^113^	DBCO-ZnPc-LP	808 nm, 0.1 W·cm^−2^, 5 min	PA image-guided PTT/PAT	• Strong heat energy transfer for PTT
				• Thermal-enhanced ultrasonic shockwave for PAT
Ding *et al.*^57^	FA-ZnPcNDs	808 nm, 0.5 W·cm^−2^, 3 min	PA image-guided PDT	• Excellent water solubility and stealth properties
Li *et al.*^116^	NanoPcTBs	655 nm, 2.0 W·cm^−2^, 1 min. + 0.22 W·cm^−2^, 5 min	PA/FL image-guided PTT/PDT	• Activatable in biotin receptor-positive cancer cells
				• Intrinsic PA+PTT properties
				• Cancer-specifically activating FL+PDT properties
Mauriello-Jimenez *et al.*^122^	BSPOR, BSPHT	750 nm, 800 nm, 3 W, 1.57 s	PA image-guided PDT	• Two-photon excited PDT for precise treatment in deep tissues.

## IV. CONCLUSIONS AND OUTLOOK

In this review, we summarized recent advances in nanoformulated Pc/Nc agents for PA imaging, synergetic biomedical imaging, biochemical sensing, and phototherapy. Due to strong extinction coefficients at long wavelengths ( $\varepsilon_{\text{max}}$ > 10^5^ M^−1^ cm^−1^, $\lambda_{\text{max}}$ > 650 nm), Pc/Ncs inherently possess high contrast for PA imaging in deep tissues. Diverse metal chelation can also be performed to modulate their optical properties to further increase the extinction coefficients (1) for high-sensitive PA imaging, (2) to enable multiplexing imaging by sharpening and separating the absorption peak, and (3) to explore multimodal imaging such as^64^Cu PET imaging. A large variety of chemical substituents have been reported in the literature, which can easily conjugate on Pc/Nc peripheries and induce targeting ability, switchable/activatable properties, enhanced stability, and improved water solubility. Many Pc/Ncs have also been used as PA image-guided phototheranostic agents in addition to their conventional use as photosensitizers in phototherapy. Rich absorption over NIR-I window has well verified its suitability as an angiographic contrast agent, and breaking its barrier to NIR-II window has overlooked its application to reveal unseen physiological findings from important deeply located organs as (1) gastrointestinal tract, (2) lymphatic system, and (3) excretory system. Intestinal motility disorder is one of the common factors that may deteriorate to severe bacterial infection and diabetes.^125^ Sentinel lymph node biopsy is a decisive process to staging of metastatic cancer and plays a critical role in future treatment and prognosis.^126^ Venous pyelogram is a test to check for abnormalities in kidney function, stones in the kidneys and ureters, and cancer.^127^ While application sites are inaccessible with noninvasive optical imaging techniques, aforementioned diagnostic protocols involve incision or cumbersome minimal invasive catheterized/endoscopic technique, otherwise heavily rely on ionizing radiation procedures as X-ray and computed tomography. As being a noninvasive, nonionizing process, NIR-II PAI possess high functional potential to innovate conventional diagnostic protocol of unreachable organs. Strong NIR absorption and photostability are their true strengths as multi-modal bioimaging and phototherapy agents. To achieve successful clinical translation and commercialization, rigorous and systemic studies must precede the approval showing further improvements in biocompatibility, solubility, clearance, and diagnostic/therapeutic efficacy. With the current emerging trends and continuous developments, we believe Pc/Ncs could serve as a vital tool for biomolecular imaging and theranostics in the near future.

## AUTHORS' CONTRIBUTIONS

E.Y.P. and D.O. contributed equally to this work.

## Data Availability

Data sharing is not applicable to this article as no new data were created or analyzed in this study.
